# Analysis of the CCR3 promoter reveals a regulatory region in exon 1 that binds GATA-1

**DOI:** 10.1186/1471-2172-6-7

**Published:** 2005-04-04

**Authors:** Nives Zimmermann, Jessica L Colyer, Laura E Koch, Marc E Rothenberg

**Affiliations:** 1 Division of Allergy and Immunology, Cincinnati Children's Hospital Medical Center, Cincinnati, Ohio, USA; 2 Department of Pediatrics, University of Cincinnati College of Medicine, Cincinnati, Ohio, USA

## Abstract

**Background:**

CC Chemokine Receptor 3 (CCR3), the major chemokine receptor expressed on eosinophils, binds promiscuously to several ligands including eotaxins 1, 2, and 3. Even though the only cells that consistently accumulate following eotaxin administration *in vivo *are myeloid cells (primarily eosinophils), other cell types have recently been shown to express CCR3. It is therefore important to elucidate the molecular mechanisms regulating receptor expression.

**Results:**

In order to define regions responsible for CCR3 transcription, a DNAse hypersensitive site was identified in the vicinity of exon 1. Coupled with our previous data implicating exon 1 in CCR3 transcription, we hypothesized that transcription factors bind to exon-1. Electrophoretic mobility shift analysis revealed that nuclear proteins in eosinophilic cells bound to exon 1. Furthermore, antibody interference and mutation studies demonstrated GATA-1 binding to exon 1. In order to test the 1.6-kb CCR3 promoter element (that includes exon 1) for *in vivo *function, this region was used to generate transgenic mice that expressed a reporter protein. Strong transgene expression was achieved, with the pattern of expression suggesting a broad acting promoter.

**Conclusion:**

The transcription factor GATA-1 binds to CCR3 exon 1. The 1.6-kb CCR3 promoter element, that includes exon 1, is a strong promoter *in vivo*.

## Background

CCR3, the eotaxin receptor, is the major chemokine receptor expressed on eosinophils, basophils and a subpopulation of Th2 lymphocytes [1-10]. Recently, CCR3 has been shown to be upregulated on neutrophils and monocytoid U937 cells by interferons *in vitro *and to be expressed by endothelial cells, epithelial cells and mast cells [11-16]. The relevance of these findings and the function of CCR3 on non-leukocytes remain to be elucidated since the only cells that consistently accumulate following eotaxin administration *in vivo *are myeloid cells (primarily eosinophils) [17-20].

To date, the complete mRNA and genomic organization of only a limited number of chemokine receptors has been described [21-26]. These studies have shown that the 5' untranslated region (5'UTR) can be complex and contain up to 11 exons as in the CXCR2 gene. As a result, alternative splicing and transcription directed by multiple promoters can give rise to variable mRNA isoforms. The function of these 5' untranslated exons has not been examined except for a single study focused on CCR2, demonstrating a transcriptional role for exon 1 [24]. We have previously characterized the genomic structure and promoter function of the human CCR3 gene [27]. The CCR3 gene contains at least 4 exons that give rise to multiple mRNA species by alternative splicing. The first 1.6 kb of the 5' flanking region of exon 1 had strong promoter activity in eosinophilic, lymphoid and respiratory epithelial cell lines. Deletion analysis revealed differential regulation of the CCR3 promoter in eosinophilic and epithelial cell lines suggesting the presence of lineage-specific elements. Interestingly, exon 1 enhanced the activity of the promoter. Since our initial characterization, two other groups have studied the CCR3 promoter [28,29]; however, their studies focused on lymphocytic and monocytic cell lines, respectively, rather than eosinophils. Scotet et al. [29] demonstrated that the human CCR3 promoter is active *in vitro *in lymphocytic cell lines. They also demonstrate a role for chromatin remodeling in the regulation of CCR3 expression in Th2 cells. Vijh et al. [28] demonstrated that the human CCR3 promoter is active in monocytic cell lines and defined the minimal promoter that consists of a downstream promoter element (DPE), a common element in Drosophila genes, but rare in human genes. This element is upstream (50 bp) of the exon 1 sequence studied in the current report.

It has been reported that 5' untranslated exons, and sometimes introns, can regulate the expression of genes in two different ways. An untranslated sequence can act as a tissue-specific translational regulator. A striking example is the gonadotropin releasing hormone gene which is transcribed in multiple tissue, but cannot be translated due to the lack of specific intron removal [30]. Alternatively, untranslated regions (UTR) can facilitate transcription of a gene. Examples include a GATA-1 site in the 5'-UTR of the γ-globin gene, an HNF-1 site in the plasminogen gene and a C/EBP site in the CCR2 gene [24,31,32]. While the mechanism of action is not completely clear, it is thought that transcription factors binding to untranslated regions affect transcription of the gene through interactions with the RNA transcription complex. The role of untranslated exons in the CCR3 gene has not been studied. In this report, DNase I hypersensitivity identified a major hypersensitive site located in the vicinity of untranslated exon 1. Furthermore, the transcription factor GATA-1 is shown to bind to untranslated exon 1, suggesting a potential mechanism for the regulation of CCR3 transcription by this exon. Finally, using a transgenic approach, we demonstrate that the 1.6 kb 5' flanking region of CCR3 (including exon 1) has promoter activity *in viv*o.

## Results

### DNase I hypersensitivity in the CCR3 locus

**Figure 1:**
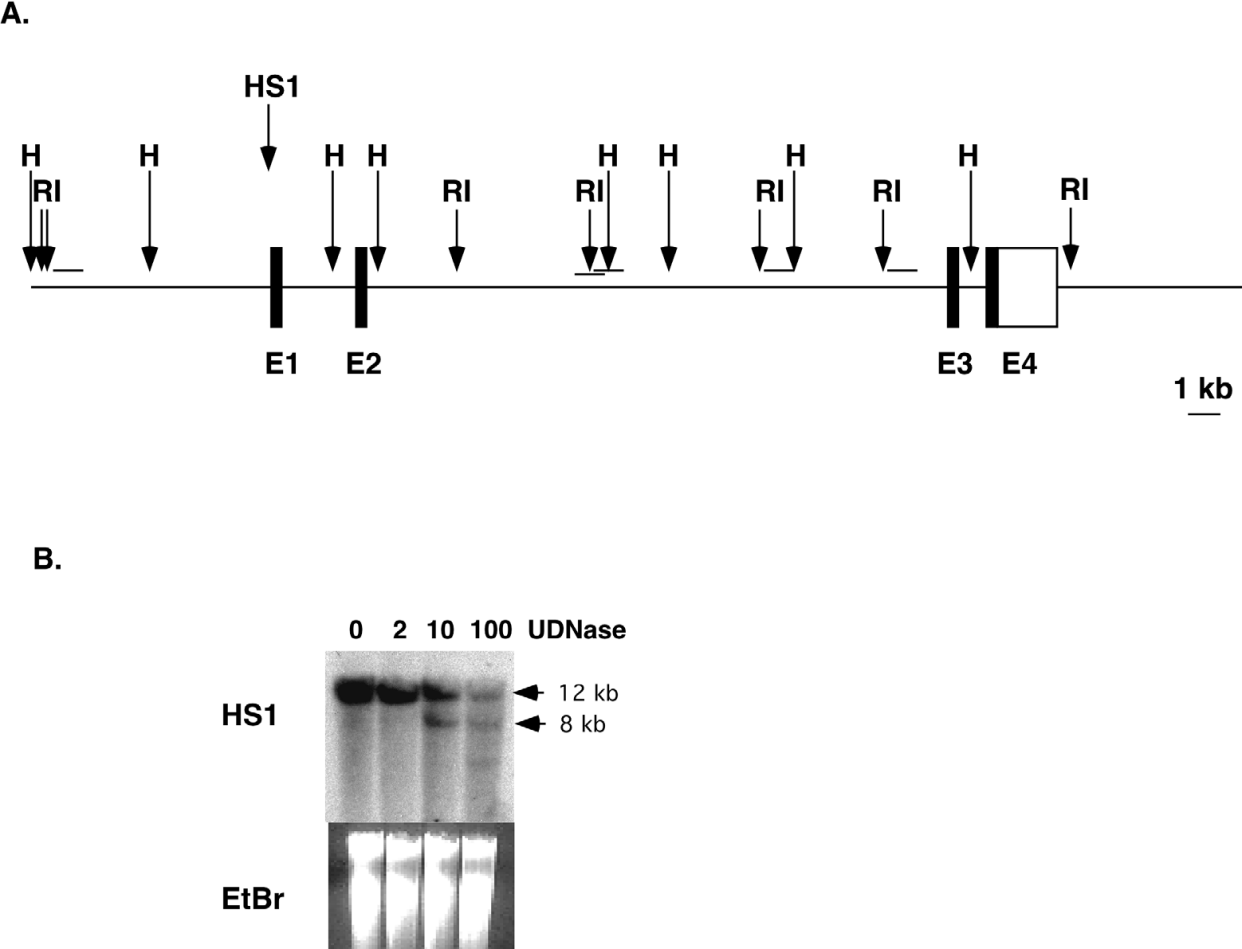
**DNase I hypersensitivity studies. **In (A), a schematic representation of the CCR3 gene is shown. Exons 1 through 4 (E1 – E4) are depicted as boxes; open box represents the open reading frame, while the closed box represents the untranslated region. The position of Hind III (H) and Eco RI (RI) restriction sites is depicted with arrows and probes used for DNase I hypersensitivity are depicted as lines above the genomic fragment. The position of hypersensitive site is depicted as HS1. In (B), Southern blot analysis for the HS site is shown. Nuclei from the primary human eosinophils were digested with indicated doses of DNase I for 5 minutes at 30°C. Following DNA purification and digestion with restriction enzymes (shown in figure is Eco RI), DNA was electrophoresed on an agarose gel and transferred to nylon membranes. Following hybridization with genomic probes, membranes were exposed to film. Size markers are shown on the right. Ethidium bromide staining of the gel is also shown.

We aimed to define regulatory regions in the CCR3 gene by a gene-wide search. Thus, we performed DNase I hypersensitivity of CCR3 in primary human eosinophils. The entire 24 kb of the CCR3 gene were screened for DNase I hypersensitivity using probes specific for Eco RI and Hind III fragments (Figure 1A and data not shown). Only one hypersensitive site was noted; this was in the area of exon 1 (Figure 1B). Similar results were obtained with nuclei from the eosinophilic AML14.3D10 cell line (data not shown). This cell line does not express CCR3 unless induced with butyric acid and IL-5; suggesting that chromatin remodeling in the HS1 site precedes CCR3 transcription. Thus, our analysis revealed areas of active chromatin remodeling in the vicinity of exon 1 suggesting that this area may be important for CCR3 transcription.

### Binding of nuclear proteins to exon 1 of the CCR3 gene

**Figure 2:**
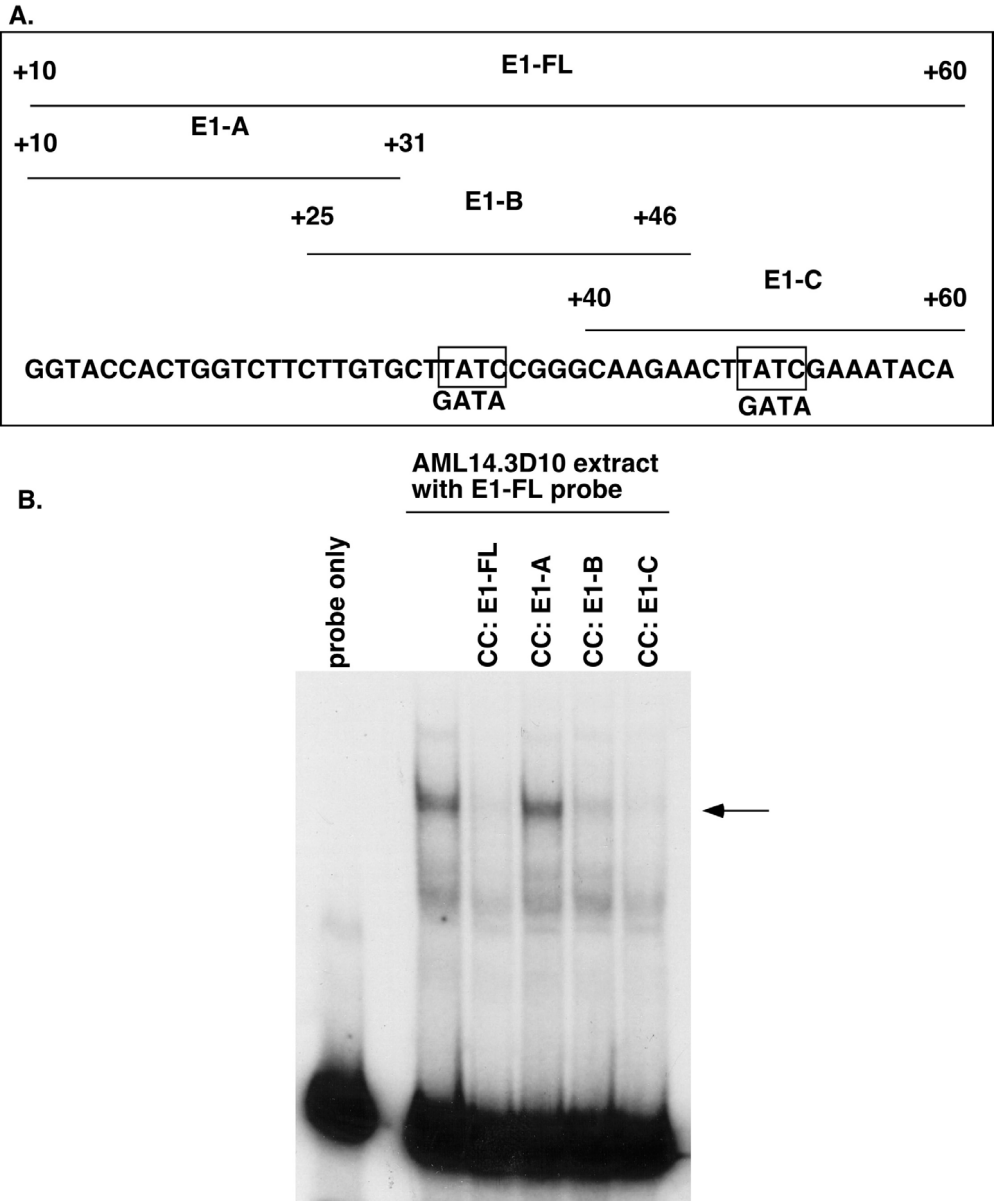
**Binding of transcription factors to CCR3 exon 1. **In (A), a schematic representation of the CCR3 exon region used for electrophoretic mobility shift assays is shown. E1-FL is the exon 1 full-length probe. Overlapping short probes are called E1-A, E1-B and E1-C, respectively. The DNA sequence is shown with GATA sites boxed. In (B), EMSA assay with the full-length probe and extracts from eosinophilic AML14.3D10 cells is shown. As cold competitors (CC), the full-length probe and short probes E1-A through C were used. The arrow depicts the specific band. A representative experiment, of three similar experiments, is shown.

DNase I hypersensitivity indicated that a region consistent with exon 1 is active in CCR3 transcription. Together with our previous data showing that untranslated exon 1 has an important role in CCR3 transcription [27], we hypothesized that nuclear proteins bind to exon 1, and in turn regulate the transcription of CCR3. In order to test this hypothesis, a double-stranded oligonucleotide probe that corresponds to bp +10 to +60 of the CCR3 gene was prepared, referred to as E1-FL (exon 1- full length, Figure 2A). This is the exact sequence that was deleted in the CCR3(-exon1).pGL3 plasmid that demonstrated decreased activity compared to the full length 1.6 kb construct [27]. Nuclear extracts from AML14.3D10 cells were incubated with the probe and resolved on a polyacrylamide gel. Two bands were visible (Figure 2B). The upper band was eliminated when 150x molar excess of the unlabelled probe was used (CC: E1-FL in Figure 2B), indicating that this is the specific band. In order to precisely localize the region responsible for factor binding, overlapping cold competitors were used: E1-A spanning from +10 to +31, E1-B spanning from +25 to +46 and E1-C spanning from +40 to +60. The specific band was eliminated with E1-B and E1-C cold competitors indicating that the factor binds in the region between +25 and +60 (Figure 2B). In summary, these data indicate the presence of proteins in the nuclei of AML14.3D10 cells that bind to CCR3 exon 1 between bp 25 and 60.

### Binding of GATA-1 to exon 1 of the CCR3 gene

**Figure 3:**
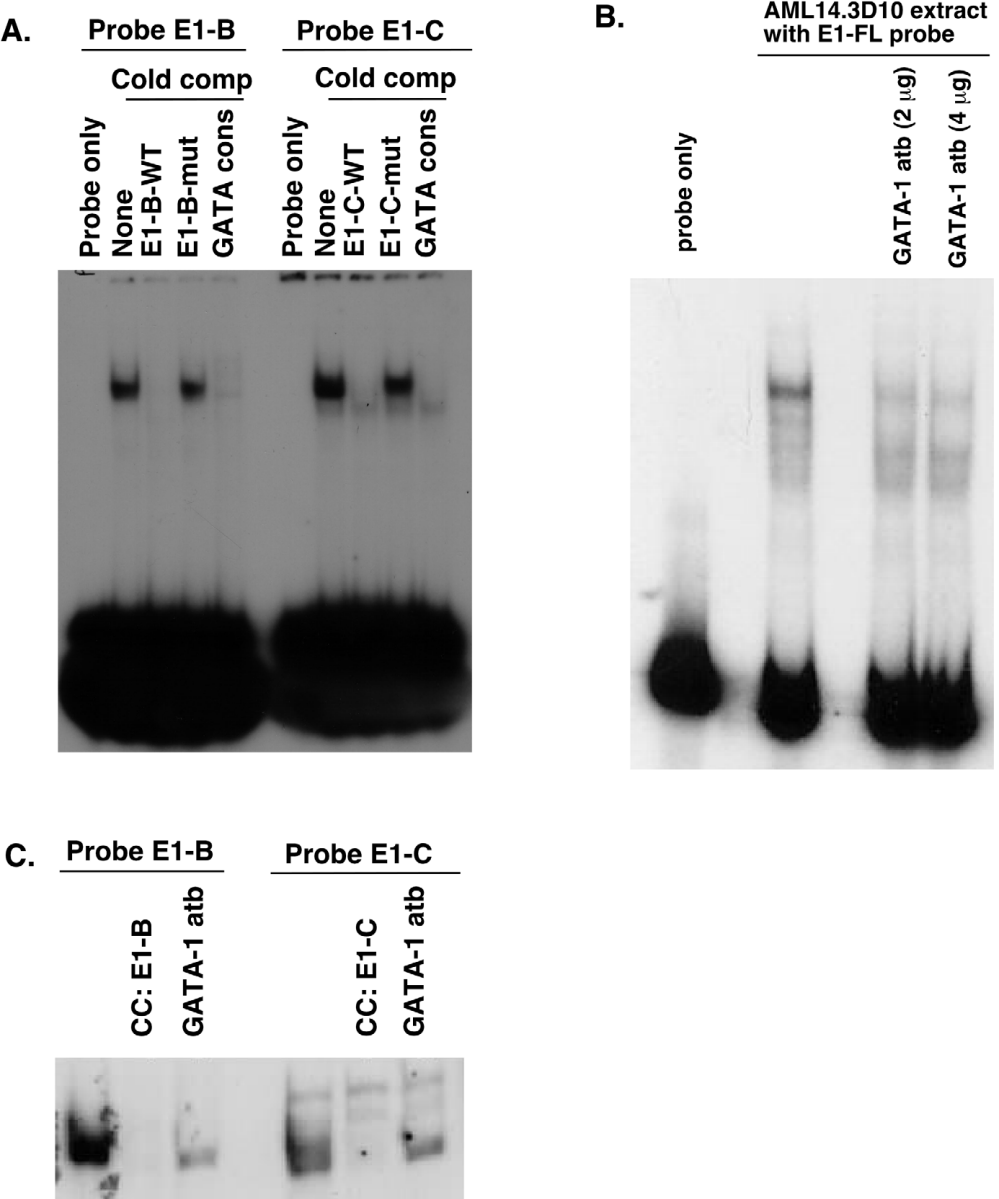
**Binding of GATA factors to CCR3 exon 1. **In (A), EMSA assay with probes E1-B (left panel) and E1-C (right panel) and nuclear extracts from AML14.3D10 cells is shown. Three cold competitors were used in each case: the probe itself (E1-B-WT and E1-C-WT, respectively), the probe with the GATA site mutated (E1-B-mut and E1-C-mut, respectively) and the GATA consensus oligonucleotide (GATA cons). A representative experiment, of three similar experiments, is shown. In (B), EMSA assay with the full-length probe (E1-FL) and extracts from eosinophilic AML14.3D10 cells in the presence or absence of antibodies against GATA-1 is shown. The arrow depicts the specific band. In (C), EMSA assay with probes E1-B (left panel) and E1-C (right panel) and nuclear extracts from AML14.3D10 cells and cold competitor probe itself (CC: E1-B and CC: E1-C, respectively) or anti-GATA-1 antibody (GATA-1 atb) is shown.

In order to define the proteins capable of binding to CCR3 exon 1, the exon 1 sequence was analyzed using the publicly available TFSEARCH engine. This analysis indicated the presence of consensus DNA-binding sites in the exon 1 region for several transcription factors (i.e. GATA, AML1, SRY, S8 etc.). Proteins of the GATA family have been detected in eosinophils (specifically GATA-1, GATA-2 and low levels of GATA-3) [33]. Additionally, GATA-1 transactivates the EOS47 promoter, an eosinophil-specific promoter, through a site in the 5'UTR [34]. Therefore, we hypothesized that proteins of the GATA family bind to sites in exon 1. In order to address the binding of GATA proteins to CCR3 exon 1, we prepared oligonucleotides corresponding to probe E1-B and -C with the GATA site mutated. As demonstrated in Figure 3A, when used as cold competitor, the GATA mutant oligonucleotide was not capable of interfering with the binding of nuclear factors to either CCR3 exon 1 probe E1-B or probe E1-C. Additionally, we used a GATA consensus oligonucleotide with binding sites for GATA proteins. This oligonucleotide completely abolished binding of nuclear factors to both CCR3 exon 1 probes E1-B and E1-C (Figure 3A). Together, these data indicate that GATA proteins, present in nuclear extracts of AML14.3D10 cells, are capable of binding to CCR3 exon 1 sequence.

In order to delineate which GATA factors are binding to exon 1, nuclear extracts from AML14.3D10 cells were incubated with the radiolabelled full-length CCR3 exon 1 probe in the presence or absence of anti-GATA-1 antibody. As seen in Figure 3B, the GATA-1 antibody was able to interfere with the binding of the protein factor from the nuclear extracts to the radiolabelled probe. In order to more precisely localize to which part of exon 1 GATA-1 is capable of binding, overlapping oligonucleotide probes were radiolabelled and incubated with nuclear extracts from AML14.3D10 cells in the presence and absence of the GATA-1 antibody. This treatment efficiently inhibited the binding of probes E1-B and -C to the nuclear factor (Figure 3C). These data indicate that GATA-1, present in nuclear extracts of AML14.3D10 cells, is at least one of the factors capable of binding the CCR3 exon 1 between bp 25 and 60.

### Characterization of the CCR3 promoter in vivo

**Figure 4:**
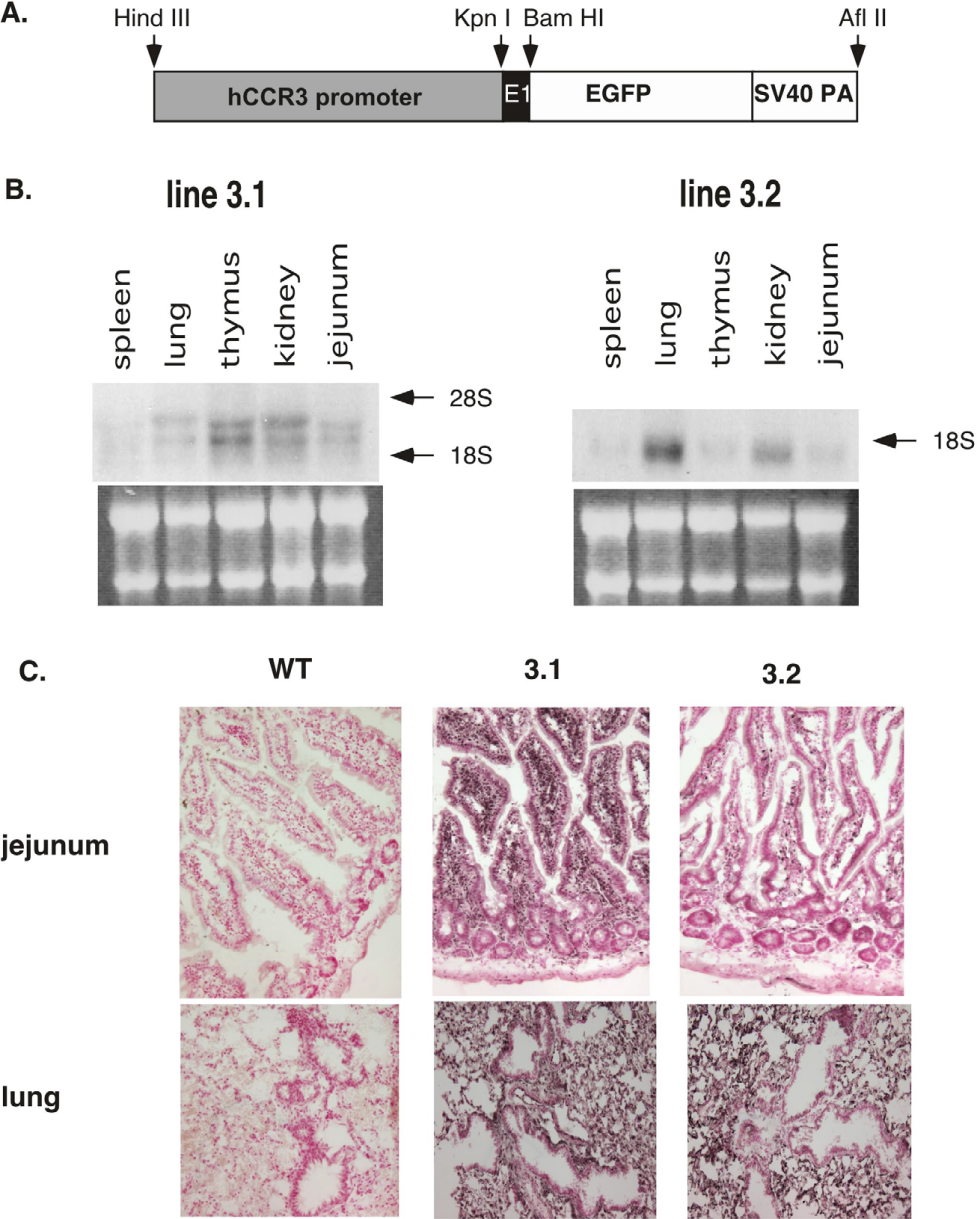
**The CCR3/EGFP transgenic mice. **In (A), a schematic representation of the transgenic construct is shown. The genomic fragment encoding 1.6 kb of the CCR3 promoter including 60 bp of exon 1 (E1), was cloned into the Hind III and Bam HI sites upstream from the EGFP gene in the pEGFP1 vector. The positions of restriction sites are indicated. In (B), Northern blot analysis of multiple tissues from transgenic mice is shown. RNA was isolated from the spleen, lung, thymus, kidney and jejunum of CCR3/EGFP transgenic mice (lines 3.1 and 3.2). 10 μg total RNA was electrophoresed on an agarose gel, transferred to nylon membranes and probed with the SV40 polyA probe in order to detect expression of the transgene. Position of 28S and 18S is depicted with arrows. Ethidium bromide staining of the gel is also shown. In (C), immunohistochemistry using an anti-GFP antibody is shown. Organs were collected, frozen sections obtained and anti-GFP immunohistochemistry performed. Positive staining is represented as black precipitate.

We have previously characterized the human CCR3 promoter *in vitro *[27]. In order to determine if this region had promoter activity *in vivo *and to assess cell-specificity, we generated transgenic mice expressing the reporter gene EGFP under the control of the CCR3 promoter (Figure 4A). 1.6 kb of the promoter and 60 base pairs of exon 1 were cloned upstream of the EGFP gene. Seven transgenic founder lines were identified. Two of the founder lines did not transmit the transgene to their offspring and one line did not show transgene expression as determined by Northern blotting and RT-PCR (data not shown). In four of the lines the level and pattern of transgene expression varied suggesting integration site effects. Two of the lines (4.1 and 4.2) displayed minimal mRNA expression and were not further analyzed. In contrast, the other two lines (3.1 and 3.2) showed high expression of the transgene in multiple tissues. Line 3.1 displayed varying levels of mRNA expression in all organs tested: lungs, kidneys, jejunum, thymus, bone marrow, and spleen, with highest expression in the thymus (by Northern blot analysis, Figure 4B and RT-PCR, data not shown). While we expected the transcript to be about 1 kb in size (Figure 4A), two bands ~4 and 5 kb were apparent. Since this was not the case with other lines, we suspect it is integration-site specific and another gene may have been transcribed together with GFP. However, the size of the RT-PCR amplified fragment was of the expected size and the protein was detectable by immunohistochemistry (see below) thus implying that this did not affect the translation of the protein and integrity of the epitope for the antibody. Expression in the lungs, jejunum, thymus and kidney was confirmed by anti-GFP immunohistochemistry (Figure 4C and data not shown). In the jejunum, staining was present predominantly in stromal cells (Figure 4C). Of note, eosinophils normally reside in the gastrointestinal tract and are located in the stroma of the jejunum. However, in the lungs, which are normally devoid of eosinophils at baseline, there was GFP expression that was confined to the alveolar lining consistent with predominant expression in type I pneumocytes (Figure 4C). In the thymus, compared to staining without primary antibody (data not shown), all thymocytes stained positive for GFP. Additionally, in the kidney all cells were positive, both in the medulla and cortex (data not shown). Line 3.2 displayed a different pattern of transgene expression. Northern blot analysis revealed expression in multiple organs with the lungs and kidneys expressing the highest levels (Figure 4B). Consistent with this, protein was detected by immunohistochemistry at high levels in lungs and kidneys and only marginally in the jejunum (Figure 4C and data not shown). While staining in the lungs was comparable to line 3.1, only a minority of stromal cells and no epithelial cells were stained in the jejunum. In the kidney, almost all cells expressed the transgene in the medulla, while in the cortex staining was prominent in the glomeruli. There was no anti-GFP staining observed in wild type mice (Figure 4C). No changes in expression were found when the transgene was crossed with CD2.IL-5 transgenic mice (data not shown). In summary, the 1.6 kb of the 5' flanking region of the CCR3 gene has strong promoter activity *in vivo*. However, both the level and pattern of expression vary between founder lines and lack cell specificity. Thus, the identified region of the CCR3 promoter contains a broadly active promoter with hematopoietic and non-hematopoietic activity.

## Discussion

In this report, DNase I hypersensitivity implicated untranslated exon 1 in regulating CCR3 transcription. Furthermore, nuclear proteins derived from eosinophilic cells were shown to bind CCR3 exon between nucleotides +25 to +60. Using unlabelled competitors and antibodies, proteins of the GATA family, specifically GATA-1, were shown to bind to this region. Taken together, these data suggest that untranslated exon 1, via GATA-1, has a regulatory role in CCR3 transcription. Finally, we demonstrate that the 1.6-kb CCR3 promoter element, that includes exon 1, is broadly active *in vivo*.

The HS1 site was located in the vicinity of exon 1. Combined with our previous results demonstrating diminished promoter activity when exon 1 is deleted from the promoter construct, these data suggested that 5' untranslated exon 1 may have a regulatory function. It has been reported that the 5' untranslated exons may contain sequences that facilitate transcription of the gene. Examples include a GATA-1 site in the 5'-UTR of the γ-globin gene, an HNF-1 site in the plasminogen gene, a PU.1 site in the PU.1 gene and a C/EBP site in the CCR2 gene [24,31,32,35]. Thus, we hypothesized that nuclear proteins bind to exon 1. Our EMSA analysis, coupled with cold competitors and specific antibodies, indicates that proteins of the GATA family, specifically GATA-1 bind to the CCR3 exon 1. Thus, GATA-1 binding to exon 1 may regulate CCR3 transcription. Alternatively, GATA-1 binding to exon 1 may affect transcription start site function, RNA stability or translation. These possibilities will be addressed in future studies.

It is important that this data be viewed with what is known about other myeloid-specific promoters, that have often proven to be difficult to function independently *in vivo*. For example, constructs using the 5' flanking region of myeloid-specific genes have not been useful for transgenic work (such as the CD14 promoter [36], the c-kit promoter [37], or the 1.7 kb CD11b promoter [38]). Better success was obtained when the entire gene, including the open reading frame, was used (e.g. the human cathepsin G, chicken lysozyme and c-fps/fes transgenic constructs [39-41]). These constructs were at least 6 kb in size and contained all exons and introns and several kb of 5' and 3' flanking sequence. Presumably, these larger constructs contained the locus control region (LCR)- sequences that have the ability to dominantly control gene expression in any chromosomal region. This in turn allows for a high degree of consistency among independent mouse lines with regard to cell specificity, level of expression and proportionality to gene copy number. These regions may be located at several different sites in the gene, including introns and coding exons; thus, screening with DNase I hypersensitivity is usually the first method employed to identify these regions. Transgenic mice expressing the EGFP reporter gene under the control of the CCR3 promoter demonstrate that the 1.6 kb promoter and 60 bp of exon 1 of the CCR3 gene confer strong promoter activity *in vivo*. However, these sequences do not contain the entire LCR, since expression of the reporter gene was variable among multiple founder lines. DNase I hypersensitivity studies discovered one hypersensitive site in the CCR3 locus. The HS1 site was apparently not sufficient for integration-site independent effects, since it is contained in the promoter construct used. Thus, future studies will need to broaden the search for the CCR3 LCR. It is important to note that the true cellular specificity of CCR3 has not been established. While this gene product is often considered to be specific for inflammatory cells involved in allergic inflammation (eosinophils, mast cells, and possibly Th2 cells), several reports have documented expression by other cell types including additional leukocytes (e.g. dendritic cells), as well as tissue cells (epithelial and endothelial cells) [11-16,42]. Thus, it remains to be determined if our observation that the CCR3 promoter has broad activity *in vivo *represents the true endogenous activity.

Since CCR3 is expressed strongly on eosinophils, analysis of the signals that induce its expression may give insight into the molecular mechanisms for the commitment of myeloid progenitors to the eosinophil lineage. It is generally believed that transcription factors are the final common pathway driving differentiation and that hematopoietic commitment to different lineages is driven by alternative expression of specific combinations of transcription factors [43,44]. Although no eosinophil-specific transcription factors have been reported, eosinophil commitment appears to be regulated by GATA-1, PU-1 and C/EBP proteins [34,45-48]. Consistent with this, DNA binding sites for these transcription factors are found in several eosinophil-selective promoters, such as the promoter for major basic protein (MBP), IL-5 receptor alpha (IL-5Rα) chain and Charcot-Leyden crystal (CLC) protein. Specifically, ectopic overexpression of GATA-1 in chicken myeloblasts leads to transdifferentiation into eosinophils or thromboblasts depending on the dose used [47]. GATA-1 transactivates several eosinophil-selective promoters [48] including the avian EOS47 promoter in which the GATA-1 site is located downstream of the transcription start site [34].

## Conclusion

In summary, in this report we have demonstrated that: 1) DNase I hypersensitivity studies implicate untranslated exon 1 in CCR3 transcription; 2) proteins of the GATA family, specifically GATA-1, bind to untranslated exon 1 in the CCR3 gene; and 3) the 1.6 kb 5' flanking region of the CCR3 gene is broadly active as a promoter *in vivo*.

## Methods

### Cell culture

The AML14.3D10 cell line (kindly provided by C.C. Paul, Dayton VA Medical Center, Dayton, OH) [49,50] was grown in RPMI 1640 (Gibco BRL, Gaithersburg, MD) containing 10% fetal calf serum (FCS, Gibco BRL), 50 μM 2-mercaptoethanol (Sigma, St. Louis, MO), 0.1 mM nonessential amino acids (Gibco BRL), 1 mM sodium pyruvate (Sigma), and penicillin-streptomycin (Gibco BRL). Eosinophils were isolated by anti-CD16 negative selection from granulocyte preparations of healthy or atopic volunteers as described previously [51].

### DNase I hypersensitivity

Nuclei were derived from cell lines and primary cells using a polyamine buffer containing 0.34 M sucrose, 13.3 mM Tris (pH7.5), 53.2 mM KCl, 13.3 mM NaCl, 2 mM EDTA, 0.5 mM EGTA, 0.133 mM spermine, 0.5 mM spermidine, 0.1% TritonX-100, 2 mM MgCl_2 _and freshly prepared 2-mercaptoethanol and phenylmethylsulfonyl fluoride (PMSF). Nuclei were then centrifuged at 2300 g for 30 min over a cushion of 1.2 M sucrose and washed twice prior to resuspension in DNase I digestion buffer (60 mM KCl, 5 mM MgCl_2_, 15 mM Tris (pH7.5), 0.1 mM EGTA, 0.5 mM DTT and 5% glycerol). Nuclei were resuspended at a concentration of 2–4 × 10^6 ^nuclei/ml and gentle DNase I digestion was carried out in a volume of 0.2 ml with 0 to 100 units of DNase I (Roche) for 5 minutes at 30°C. Following DNase I treatment, nuclei were lysed and DNA purified by phenol/chloroform extraction and ethanol precipitation. DNA was digested with appropriate restriction enzymes (Eco RI and Hind III) and electrophoresed on a 0.8% agarose gel. Following transfer to nylon membranes, hybridization was performed using standard procedures. Probe fragments were made by PCR from genomic DNA. Figure 1 depicts the CCR3 gene structure and restriction fragments and their corresponding probes that were used to span the entire gene.

### Preparation of nuclear extracts from cultured cells

Cultured cells were washed twice with ice cold phosphate buffered saline (PBS, Gibco BRL). 2.5 × 10^6 ^cells were lysed in lysis buffer [100 mM *N*-2-hydroxyethylpiperazine-*N*'-2-ethanesulfonic acid (HEPES), pH 7.9, 10 mM KCl, 0.1 mM EDTA, 1.5 mM MgCl_2_, 0.2% Nonidet P-40, 1 mM dithiothreitol (DTT), and 0.5 mM PMSF], briefly vortexed at a moderate speed, then incubated on ice for 5 minutes. Sample was centrifuged and the pellet was next resuspended in 20 μl of extraction buffer (20 mM HEPES, pH 7.9, 420 mM NaCl, 0.1 mM EDTA, 1.5 mM MgCl_2_, 25% glycerol, 1 mM DTT, and 0.5 mM PMSF), mildly vortexed, and incubated on ice for 15 minutes. Samples were centrifuged to pellet the nuclear debris. Supernatants were placed in silicon coated microcentrifuge tubes and stored at -80°C until further use.

### Synthetic oligonucleotides

Single-stranded oligonucleotides based on the sequence of untranslated exon 1 of the CCR3 promoter were synthesized by Integrated DNA Technologies, Inc. (Coralville, IA). One full length 51 base pair (+10 to +60) oligonucleotide (GGTACCACTGGTCTTCTTGTGCTTATCCGGGCAAGAACTTATCGAAATACA) and three overlapping oligonucleotides (+10 to +31, +25 to +46, and +40 to +60) and their reverse complements were produced (Figure 2). Two of the overlapping fragments contained putative GATA binding sites (underlined). Two mutants and their complements were also made which changed the GATA sequence TATC to TTGA. This mutation does not change the GC content of the oligonucleotides, nor does it create a new transcription factor site for any of the transcription factors represented in the publicly available TFSEARCH engine. Each oligonucleotide was resuspended to a concentration of 50 μM in TE buffer (10 mM Tris-Cl, pH 7.4, and 0.1 mM EDTA, Sigma). Complimentary single-stranded oligonucleotides were annealed at a concentration of 10 mM in restriction enzyme buffer M (10 mM Tris-Cl, 10 mM MgCl_2_, 50 mM NaCl, and 1 mM dithioerythritol, Roche Molecular Biochemicals, Indianapolis, IN). Samples were placed in a 95°C dry heat block for 5 minutes and then the block was removed from the unit and allowed to cool slowly to room temperature. The double-stranded oligomers were diluted to 1 μM with TE buffer and 30 ng were end-labelled using [γ-^32^P]ATP (NEN Life Science, Boston, MA) and T4 polynucleotide kinase (Gibco BRL). Probe was purified over Quick Spin G-25 Sephadex columns (Roche) and recovered in a volume of 50 μl.

### Electrophoretic mobility shift assay (EMSA)

Protein content of the nuclear extracts were determined by Bradford (Coomassie) assay (Pierce, Rockford, IL). Total protein (5 μg) was incubated on ice for 10 minutes with 2X EMSA buffer (24% glycerol, 0.08 μg/ml poly dI-dC, 24 mM HEPES, pH 7.9, 8 mM Tris-Cl, pH 7.9, 2 mM EDTA, 2 mM DTT, 50 mM KCl, and 10 mM MgCl_2,_), and when indicated, with 150 fold excess of cold competitor oligonucleotide. Radiolabelled oligo probe was added to each sample and incubation continued for an additional 10 minutes on ice. For antibody supershift assays, anti-GATA-1 antibody (clone C20, Santa Cruz Biotechnology, Santa Cruz, CA) was added following the addition of the probe and samples incubated on ice for one hour. In control experiments, isotype-control antibodies did not have a significant effect. The DNA-protein complexes were then resolved on a non-denaturing 5% acrylamide gel [29:1 acrylamide/bis-acrylamide, 0.5X TBE buffer (44.5 mM Tris, 44.5 mM borate, and 1 mM EDTA), and 25% glycerol] at constant current of 30 mA for approximately 60 minutes. Gels were dried on blotting paper and exposed to x-ray film.

### Generation and analysis of transgenic lines

1.6 kb of the CCR3 promoter, including 60 bp of untranslated exon 1, was subcloned into the pEGFP vector (Clontech, Palo Alto, CA). The transgenic construct containing the promoter, reporter gene enhanced green fluorescent probe (EGFP) and the SV40 polyadenylation signal (Figure 4) were liberated by Afl II and Hind III digestion and injected into the pronucleus of fertilized eggs from FVB/N mice by the Transgenic Core Facility at Cincinnati Children's Hospital Medical Center. Transgenic mice were identified by Southern blot analysis after digestion with BamHI, using the SV40 poly A fragment as a probe. Lines were tested for expression of the transgene in multiple organs by Northern blotting and RT-PCT. The SV40 poly A fragment was used as a probe for Northern blotting. PCR primers for EGFP were as follows: 5'ATGGTGAGCCAAGGGCGAG3' and 5'CTTGTACAGCTCGTCCATG3'. RNA integrity and RT efficiency was verified by performing PCR for β-actin on the same cDNA samples. Primers used were as follows: 5'GGAATCCTGTGGCATCCATGAAACT3' and 5'TAAAACGCAGCTCAGTAACAGTCCG3'.

### Anti-GFP immunohistochemistry

Immunohistochemistry was performed on frozen sections essentially as described [52]. Briefly, following endogenous peroxidase quenching, slides were blocked and stained with a rabbit-anti-GFP antibody (AB3080 at 1:400 dilution, Chemicon International, Temecula, CA). The slides were washed and incubated with biotinylated goat anti-rabbit antibody and avidin-peroxidase complex (Vectastain ABC Peroxidase Elite kit, Vector Laboratories). The slides were then developed by nickel diaminobenzidine, enhanced nickel cobalt chloride to form a black precipitate and counterstained with nuclear fast red.

## Authors' contributions

JLC carried out the EMSA analysis. LEK performed the DNAse hypersensitivity studies. NZ carried out the generation and analysis of transgenic mice, participated in the design and coordination of the overall study and drafted the manuscript. MER participated in the design and coordination of the overall study and helped to draft the manuscript.
